# Exposure to selected pathogens in wild mammals from a rescue and rehabilitation center in southern Italy

**DOI:** 10.1016/j.onehlt.2025.101049

**Published:** 2025-04-21

**Authors:** A. Minichino, L. Ciuca, L. Dipineto, L. Rinaldi, S. Montagnaro, L. Borrelli, A. Fioretti, L.M. De Luca Bossa, G. Garella, G. Ferrara

**Affiliations:** a Regional Reference Center of Urban Veterinary Hygiene (CRIUV), Napoli, Italy; b Department of Veterinary Medicine and Animal Production, Federico II University of Naples, 80134 Naples, Italy; c Department of Veterinary Sciences, University of Messina, Polo Universitario dell'Annunziata, Messina 98168, Italy

**Keywords:** Reservoirs, Sentinels, Surveillance, Wildlife health, Wildlife infections

## Abstract

Wildlife is at the same time a reservoir and sentinel of numerous infections for humans and domestic animals. For this reason, wildlife rehabilitation centers represent an opportunity to carry out surveillance against the most varied infections. In this work, wild animals (canids, mustelids, erinaceids, and cervids) hospitalized at a first aid center in southern Italy were sampled and tested with multispecies ELISAs and rapid tests against a panel of pathogens, including viruses, bacteria, and parasites. Foxes and wolves were exposed to *Brucella canis*, *Coxiella burnetii*, canine coronavirus, and Pseudorabies virus. Furthermore, one and two foxes tested positive for *Anaplasma phagocytophilum* and *Dirofilaria immitis*. Although not confirmed by molecular assay, hedgehogs and porcupines tested positive for *Dirofilaria immitis* antigens. No animals were exposed to *Leishmania infantum*, *Borrelia burgdorferi*, *Mycobacterium avium*, or Schmallenberg viruses. A fox and a roe deer had antibodies against the hepatitis E virus. The overall prevalence of *Angiostrongylus vasorum* antigen was 25 % (all the positive samples were collected from red foxes). Parasitological analyses showed the positivity of wild animals to *Crenosoma* spp., *Strongyloides* spp., *Capillaria* spp., and *Cystoisospora* spp. Wild canids also tested positive for *Toxocara* spp. and *Trichuris vulpis*. The results of this study have demonstrated not only the circulation of numerous pathogens in the wildlife of southern Italy but also underlined the risk to which the operators of first aid centers are subjected, considering that some of these animals stand periods of rehabilitation even of several months.

## 1 Introduction

Infections have emerged at the animal-human interface since agriculture and farming procedures permitted people to stay in sedentary communities alongside their animals and crops [4,29]. Nevertheless, this phenomenon has grown increasingly crucial since the very start of the twentieth century, when both the magnitude and frequency of zoonotic disease outbreaks have increased [4]. Infection surveillance is a critical component of wildlife management and conservation. The frequency of wildlife-domestic animals and wildlife-human interactions has grown due to ongoing urbanization and the depletion of wild animals' habitats [4,5,27]. This proximity facilitates the interspecies spread of infections, with repercussions for all three interfaces listed above. A growing percentage of spillover conditions include wildlife [7]. The motivations and processes that enable infections to originate between wildlife and people are not fully understood, nor are the mechanisms that allow animal infections to be transferred to humans and eventually evolve into an adapted human pathogen [42]. In recent years, health and governmental authorities have become more aware of the needs of wild animals in order to protect their health [11]. Based on this principle, the establishment of wildlife rescue and rehabilitation centers occurred worldwide. These facilities serve to receive animals in distress found in a specific area and address their treatment, rehabilitation, and release back into nature [56]. This wildlife encounter also provides a unique chance to conduct infectious disease surveillance and, at the same time, accurately characterize the health status of these animals [4]. Wildlife in Italy is mainly characterized by mammals such as wild boars (which are rarely admitted to these facilities), canids (foxes and wolves), mustelids (martens, weasels, and badgers), erinaceidae (hedgehogs and porcupines), and ruminants (deer and roe deer) [47,50]. The species listed are susceptible to a wide range of infectious diseases that affect domestic animals as well as humans [31,37,65]. These animals can host several diseases, representing a threat to both human and animal health, and a sentinel that can provide critical information about the spread of infections [3]. Examples of infections that can be harbored by wildlife include viruses [like canine coronavirus (CCV), Schmallenberg virus (SBV), Paslahepevirus balayani (HEV), and Pseudorabies virus (PRV)], bacteria (like *Brucella* spp., *Coxiella burnetii*, *Mycobacterium avium*, *Anaplasma phagocytophilum*, *Ehrlichia canis*, *Borrelia burgdorferi*), and parasites (like *Toxocara* spp., *Trichuris vulpis*, *Crenosoma* spp., *Strongyloides* spp., *Capillaria* spp., *Physaloptera* spp., *Cystoisospora* spp., *Leishmania infantum*, *Dirofilaria immitis*, *Angiostrongylus vasorum*). Furthermore, in recent years, several studies have highlighted how the host range of some of these pathogens has not yet been well defined. Badgers as reservoirs for bovine TB, wild boars for PRV, and foxes for rabies are examples of wildlife that serve as reservoirs for infections. On the other hand, some bird species serve as sentinels for West Nile virus (WNV) [28,32]. Another study identified hedgehogs as possible reservoirs of Q fever [26].

The present study aimed to evaluate the exposure to different viral, parasitic and bacterial infections of wildlife hospitalized at the Wildlife Rescue Centre (CRAS) “Federico II” of the University of Naples (southern Italy), with a focus on zoonotic agents. In particular, the aim of this work was to evaluate the exposure to HEV, SBV, *C. burnetii*, PRV, CCV, *M. avium*, *B. canis*, *Leishmania*, *Anaplasma*, *Borrelia*, *Ehrlichia* as well as the antigenic positivity to *D. immitis* and *A. vasorum* and the copromicroscopic positivity to other parasites (*Toxocara*, *Trichuris*, *Crenosoma*, *Strongyloides*, *Capillaria*, *Physaloptera*, *Cystoisospora*).

## 2 Materials and methods

### 2.1 Sampling and study area

**Fig. 1:**
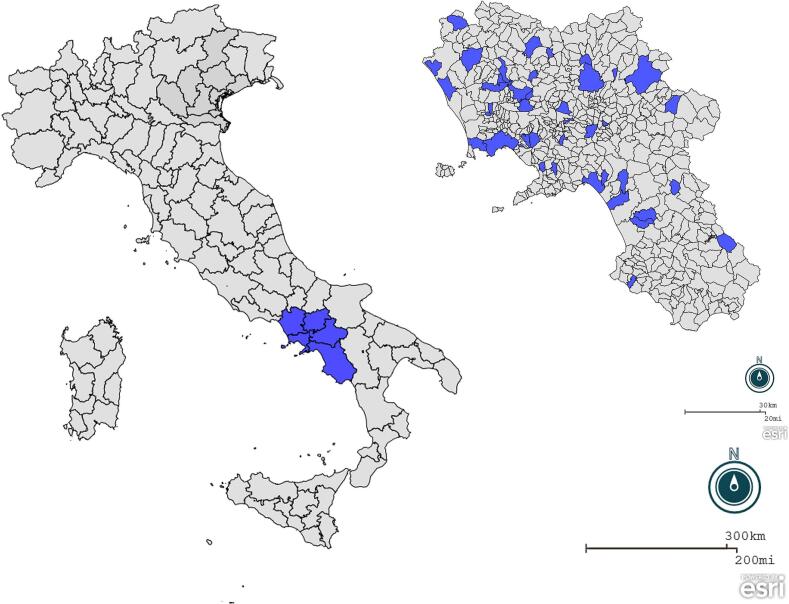
Spatial distribution of the collected samples. Fig. 1**Caption:** Origin of the sampled samples from a rescue and rehabilitation center in southern Italy: blue color indicates the sampled districts (*n* = 41).

This study was carried out in Campania, a region in southern Italy with a Mediterranean ecosystem, during the period between May 2023 and July 2024. A total of 42 animals, including 4 wolves (*Canis lupus*), 18 red foxes (*Vulpes vulpes*), 5 porcupines (*Hystrix cristata*), 4 badgers (*Meles meles*), 8 hedgehogs (*Erinaceus europaeus*), and 3 roe deer (*Capreolus capreolus*), were recovered at the Wildlife Rescue Center of the University of Naples Federico II (Italy). Since it was not possible to estimate either the number of wild populations in the Campania region or the number of animals to be rehabilitated, convenience sampling was applied. All animals belonged to the study area and were rehabilitated at the center during the sampling activities (Fig. 1), which correspond with routine diagnostic investigations (no ethical permission was required). From each animal, a blood sample was collected from the jugular and cephalic veins using a 22- and 27-gauge needle and an appropriate syringe. For a total of 20 animals (including 6 red foxes, 4 wolves, 2 badgers, and 8 hedgehogs), a fecal sample was also collected. Information regarding the precise origin (municipality), sex, age, reason for hospitalization, days of hospitalization, and release was collected and included in a supplementary file (Supplementary file 1). (See Table 1.)

**Table 1:** Diagnostic methods used in this study.

Name	Pathogen(s)	Type
SNAP 4Dx Plus (IDEXX)	*Ehrlichia*, *Anaplasma*, *Borrelia*, *Dirofilaria*	Rapid (indirect for *Ehrlichia*, *Anaplasma* and *Borrelia*; antigenic for *Dirofilaria*)
PRV/ADV gB and gE Ab Test (IDEXX)	PRV	ELISA (Competitive indirect)
ID Screen® Schmallenberg virus Competition Multi-species (ID Vet)	Schmallenberg virus	ELISA (Competitive indirect)
ID Vet ID Screen® Q fever indirect multi-species (ID Vet)	*Coxiella burnetii*	ELISA (Indirect multi-species)
ID Screen® Hepatitis E Indirect Multi-species (ID Vet)	Hepatitis E virus	ELISA (Indirect multi-species)
ID Screen® *Mycobacterium avium* Indirect Multi-species (ID Vet)	*Mycobacterium avium*	ELISA (Indirect multi-species)
Rapid serum agglutination (Rose Bengala Test, IDEXX)	Smooth *Brucella* spp.	RSA (Indirect)
*Brucella canis* IC (Agrolabo)	*Brucella canis*	Rapid (Indirect)
Canine Coronavirus Ab ELISA (Agrolabo)	Canine Coronavirus	ELISA (Indirect)
FLOTAC	*Toxocara, Trichuris, Crenosoma, Strongyloides, Capillaria, Physaloptera, Cystoisospora*	Copromicroscopic (direct)
SNAP Leishmania (IDEXX)	*Leishmania infantum*	Rapid (indirect)
Angio Detect Test (IDEXX)	*Angiostrongylus vasorum*	ELISA (direct)
Petcheck Canine Heartworm Immunoassay (IDEXX)	*Dirofilaria*	ELISA (direct)

### 2.2 Rapid and serological analysis

The blood samples were transported to the Infectious Diseases laboratory of the Department of Veterinary Medicine and Animal Production in Naples, maintaining the cold chain. Each sample was centrifuged in order to separate the serum, which, once collected, was used in the following rapid and serological tests: SNAP 4Dx Plus (IDEXX) for the detection of antibodies against *A. phagocytophilum*/*E. canis*/*B. burgdorferi*, PRV/ADV gB and gE Ab Test (IDEXX) for the detection of antibodies against PRV), ID Screen® Q Fever Indirect Multi-species (IDVet) for the detection of antibodies against *C. burnetii*, ID Screen® Schmallenberg virus Competition Multi-species (IDVet) for the detection of antibodies against SBV), ID Screen® Hepatitis E Indirect Multi-species (IDVet) for the detection of antibodies against HEV, ID Screen® *Mycobacterium avium* Indirect Multi-species (IDVet) for the detection of antibodies against *M. avium paratubercolosis* (MAP). The use of competitive ELISAs or multispecies conjugates (e.g. protein G) is beneficial since they are applicable to a wide range of species, including wild ones. However, it should be noted that the cut-offs for determining the positive or negative of the analysis may differ, affecting the final results. However, this kind of test is often employed in wild animal monitoring investigations [23,46,61]. For some species, the off-label use of the previously listed tests has been justified by other studies reported in the literature [35]. This condition reduced the diagnostic performance of the kits in some species. For example, the sensitivity and specificity of SNAP® 4Dx® Plus test in wild mammals are 80 % and 98.9 %, respectively, when compared to the McNemar's test [35]. All samples were further tested using rapid serum agglutination (RSA) by diluting the serum in rose bengale (Pourquier Rose Bengale Ag, IDEXX) as described in a previous work [48]. Wolf and fox samples were further tested for antibodies to *B. canis* and Canine coronavirus (CCV) using *Brucella canis* IC (Agrolabo) and Canine Coronavirus Ab ELISA (Agrolabo) respectively. The three roe deer samples were analyzed for the presence of antibodies against bovine herpesvirus type 1 (BHV-1) and bovine diarrhea virus (BVD) using ID Screen® IBR Indirect and ID Screen® BVD p80 Antibody Competition (IDVet). Each test was carried out and interpreted following the manufacturer's instructions.

### 2.3 Parasitological analysis

#### 2.3.1 Copromicroscopic analysis

The coprological analyses included fecal samples from red foxes, wolves, badgers and hedgehogs. All these animals were screened for helminths and protozoa using the FLOTAC dual technique with sodium chloride (specific gravity, s.g. = 1.20) and zinc sulphate (s.g. = 1.20) as flotation solutions and an analytic sensitivity of 2 eggs/oocysts/cysts/larvae per grams of faeces (EPG/OPG/CPG/LPG) [6]. In addition, the Baermann technique (for the detection of the lungworm larvae) was used only for the red foxes and wolves, due to the insufficient amount of faeces for the other species included in the study.

#### 2.3.2 2.3.1 Blood analysis

The presence of *A. vasorum* antigen in blood samples from canids was assessed using Angio Detect Test (IDEXX). *D. immitis* infection was detected using the SNAP 4Dx Plus (IDEXX), the Petcheck Canine Heartworm Immunoassay (IDEXX) and molecular analysis. SNAP Leishmania (IDEXX) was used for the detection of antibodies against L. *infantum* (other research in the literature has supported off-label application) [35].

For molecular determination, genomic DNA was extracted from 200 μl of blood, using the DNeasy® Blood and Tissue kit (Qiagen, Germany), following the manufacturer's instructions. Molecular analyses were performed following the protocols of multiplex PCR described by Rishniw et al. [62] (5.8 + ITS2 region) for simultaneous detection of *D. immitis* and *D. repens* [62].

### 2.4 Statistical analysis

Descriptive statistics were used to summarize the frequency and percentage of pathogen and parasite presence across species, sex, and age groups. This approach was selected due to the nature of the dataset, which included small sample sizes and categorical variables that limited the use of more complex statistical tests. Due to the limited sample size, no statistical inference tests such as chi-square or Fisher's were used. Descriptive analysis allowed for a clear understanding of the distribution of infections within the wildlife populations studied, providing insight into potential patterns of pathogen exposure without applying inferential statistical methods. The results are reported in terms of percentages and frequencies, highlighting the distribution of infections across different species and groups.

## 3 Results

Canine coronavirus and *B. canis* represent, respectively, the viral and bacterial pathogens to which wild canids in the Campania region are most exposed (Table 2). In fact, 5 foxes were positive for *B. canis* and 3 foxes and 2 wolves for CCV. In both cases, a seroprevalence of 22.7 % (*n* = 5) was observed. Two fox samples (4.8 %) reacted positively to RBT for the detection of anti-Brucella antibodies. Wild canids (red fox) were also exposed to *A. phagocytophilum* and *E. canis* (2.6 %, *n* = 1) (Table 3). Specific antibodies against L. *infantum*, *B. burgdorferi*, SBV, and *M. avium* were not found in any blood sample. Although susceptible, all hedgehog, porcupine, and badger samples were negative by ELISA for the detection of antibodies against HEV, *C. burnetii*, and PRV. In fact, the prevalences obtained for *C. burnetii* (2.4 %) and for PRV (7.1 % for gB and 2.4 % for gE) were attributable to seropositive canids. One fox sample reacted positively in ELISA for anti-Coxiella antibodies, two wolf samples and one fox sample reacted in competitive ELISA for PRV. Furthermore, a total of two animals (a wolf and a roe deer) presented HEV-specific antibodies (4.8 %). The three roe deer samples analyzed for the presence of antibodies against BHV-1 and BVD were negative. Specific antigens against *A. vasorum* were found in 25 % of canids (6 fox samples), while specific heartworm antigens were detected in 12.8 % of the samples tested (2 foxes, 2 hedgehogs, and 2 porcupines). Further investigations were conducted on samples of hedgehogs and porcupines, as these animals have never been described as reservoirs of *D. immitis*. However, a second, more specific ELISA and an end-point PCR method determined the negativity of these samples. In addition, two foxes had two different results for *D. immitis* antigen, as follows: positive by the SNAP test and negative by the ELISA, but negative by the PCR test in both cases, and one of them had L1 larvae of *A. vasorum* in the faeces. No faeces were available for the other animals.

**Table 2:** Surveillance about selected viruses and bacteria using serological assays (ELISA and RBT) in a wildlife rescue and rehabilitation center in southern Italy.

Species	n	HEV	SBV	*C. burnetii*	PRV gB	PRV gE	CCV	*M. avium*	*B. canis*	B. smooth
Total	42	2 (4.8 %)	0 (0 %)	1 (2.4 %)	3 (7.1 %)	1 (2.4 %)	5 (22.7 %)	0 (%)	5 (22.7 %)	2 (4.8 %)
Red foxes	18	0	0	1	1	1	3	0	5	2
Wolves	4	1	0	0	2	0	2	0	0	0
Badgers	4	0	0	0	0	0	/	0	/	0
Hedgehogs	8	0	0	0	0	0	/	0	/	0
Porcupines	5	0	0	0	0	0	/	0	/	0
Roe deer	3	1	0	0	0	0	/	0	/	0
Sex										
Male	25	0	0	1	2	0	3	0	3	1
Female	17	2	0	0	1	1	2	0	2	1
Age										
Young	17	1	0	1	0	0	2	0	5	0
Adult/Old	25	1	0	0	3	1	3	0	0	2

HEV = Hepatitis E Virus.

SBV = Schmallenberg virus.

PRV = Pseudorabies virus.

CCV = Canine Coronavirus.

**Table 3:** Surveillance about selected bacteria and parasites using rapid serological (Leishmania, Anaplasma, Borrelia, Ehrlichia) and antigenic (Dirofilaria and Angiostrongylus) assays in a wildlife rescue and rehabilitation center in southern Italy.

		*Leishmania*	*Anaplasma*	*Borrelia*	*Ehrlichia*	*Dirofilaria immitis*	*Angiostrongylus vasorum*
	**n**						
Total	39	0 (0 %)	1 (2.6 %)	0 (0 %)	1 (2.6 %)	6 (12.8 %)	6 (25 %)
Species							
Red foxes	18	0	1	0	1	2	6
Wolves	4	0	0	0	0	0	0
Badgers	4	0	0	0	0	0	/
Hedgehogs	8	0	0	0	0	2	/
Porcupines	5	0	0	0	0	2	/
Sex							
Male	24	0	1	0	1	3	6
Female	15	0	0	0	0	3	0
Age							
Young	17	0	1	0	0	1	4
Adult/Old	22	0	0	0	1	5	2

Parasitological investigations have highlighted a high prevalence of *Crenosoma* spp. (40 %) and *Capillaria* spp. (25 %) eggs described in canids and hedgehogs. *Toxocara* spp. and *T. vulpis* were detected in 25 % and 5 %, respectively, of the canid samples evaluated (Table 4). A sample of fox and badger faeces contained *Strongyloides* spp. and *Cystoisospora* spp. eggs (10 %). One badger and three hedgehog stool samples had *Physaloptera* spp. eggs (20 %). Descriptive statistics were calculated to summarize the frequency and percentage of exposure to different pathogens and parasites across species, sex, and age groups. The results highlighted that red foxes were particularly exposed to pathogens such as Canine Coronavirus (CCV), *Brucella canis*, Pseudorabies Virus (PRV), and parasites like *Toxocara* spp. and *Crenosoma* spp., suggesting that this species serves as an important reservoir in the local wildlife. Wolves also showed some exposure, though to a lesser extent. The analysis revealed a prevalence of infections such as *D. immitis* in 12.8 % of the animals and *A. vasorum* in 25 %, with higher incidence among canids, especially red foxes. However, no significant differences were observed between males and females, and the age distribution showed a slightly higher prevalence of certain pathogens, like *Dirofilaria* and *Angiostrongylus*, in younger animals. Furthermore, no animals tested positive for pathogens such as L. *infantum*, *B. burgdorferi*, or *M. avium*, indicating low exposure to these infections in the sampled species.

**Table 4:** Surveillance about selected parasites through copromicroscopic analysis in a wildlife rescue and rehabilitation center in southern Italy.

		*Toxocara*	*T. vulpis*	*Crenosoma vulpis*	*Strongyloides*	*Capillaria aerophyla*	*Physaloptera* spp.	*Cystoisospora* spp.
	**n**							
Total	20	5 (25 %)	1 (5 %)	8 (40 %)	2 (10 %)	5 (25 %)	4 (20 %)	2 (10 %)
Species								
Red foxes	6	4	1	5	1	0	/	1
Wolves	4	1	0	0	0	1	/	0
Badgers	2	/	/	0	1	0	1	1
Hedgehogs	8	/	/	3	0	4	3	0
Sex								
Male	13	4	1	6	2	2	2	2
Female	7	1	0	2	0	3	2	0
Age								
Young	9	4	1	6	1	3	2	0
Adult/Old	11	1	0	2	1	2	2	2

## 4 Discussion

In this study, exposure or positivity to different pathogens (23 in total) was evaluated in animals (*n* = 42) admitted to a wildlife rescue and rehabilitation center in the Campania region. Canids were found to be more frequently exposed to the infections tested, and in particular, they presented antibodies against CCV, HEV, *C. burnetii*, PRV, *B. canis*, and *Brucella* spp. Lower seroprevalences of approximately 5 % have been described for CCV in a small-scale study in wild canids in Southeast Brazil [8]. Antibodies against *Brucella*, and especially *B. canis*, were frequently detected in our study. Recently, outbreaks of abortion in China in blue foxes revealed exposures up to 67 % and the isolation of *Brucella melitensis* [69]. The exposure rate obtained for HEV (1/22) was in line with a study performed in Serbia where no wild canids (mainly Golden Jackals) were seropositive by ELISA, but discordant with a retrospective study performed in Tuscany, Italy, which revealed a seroprevalence of 21.5 % in red foxes [12,60]. However, it was not surprising that one out of three roe deer tested positive since wild ruminants are one of the natural reservoirs of this infection [20,68]. Although a study conducted in Chile on Darwin's foxes did not find any positive animals for *C. burnetii*, in our study, one red fox tested positive in a region endemic for Coxiellosis in ruminants (all other species were negative) [13,30].

Our findings revealed the absence of antibodies against *M. avium* and SBV in all samples. Both infections are widely distributed among domestic species in the study area (ruminants), and evidence of infection has been previously described in foxes and mustelids (up to 20 % at the molecular level) [10,18,43,44]. Positive outcomes for canine vector-borne diseases (CVBDs) were also reported in the present study (2.6 % for *E. canis* and *A. phagocytophilum*), in line with what was observed in other studies carried out in Tuscany (Italy) and the Czech Republic [12,37]. A higher prevalence of *E. canis*, however, was described in Israel (36 %) [19]. However, our study did not identify any positivity to L. *infantum* and *B. burgdorferi*, as reported in other research carried out in Italy in red foxes and European rabbits [1]. Moreover, *L. infantum* has been recently reported in wild canids in Iran (10 %), even if another study reported a seroprevalence of 0 % in Brazil [2,55]. The low prevalences observed in several studies could be due to the poor diagnostic performance of the test when used in off-label species. Although validated for other species, scientific evidence has highlighted how the tests used in this study could be useful for conducting epidemiological studies in other species as well (as wild canids, mustelids, and felids) [21,35,66].

Similar prevalences of PRV and *D. immitis* have been described in other regions of Italy [12]. Although PRV is highly lethal in domestic and wild carnivores that can become infected by coming into contact with infected pigs or meat (especially wild boar), it has been seen that in contact with low viral concentrations, animals can seroconvert without developing the disease and symptoms [9,15,17]. The discrepancy between anti-gB and anti-gE antibodies resulted from the different dynamics that characterize seroconversion against these glycoproteins in herpesvirusesand the spread of a vaccine strain (gE-deleted), able to affect carnivores, with consequent failure to seroconvert against gE [49].

The presence of *D. immitis* antigen had also been detected in porcupines and hedgehogs, but subsequent laboratory analyses based on a further antigen ELISA test and a molecular approach had excluded the presence of the pathogen. A cross-reaction with other pathogens could therefore be suspected. Recently, *D. immitis* infection has been demonstrated in badgers, causing typical cardiac alterations, with the presence of microfilaremia demonstrated by ELISA and the Knott method [41]. Considering only wild canids, the prevalence is reduced to 11 %, lower than that described in the San Miguel and Santa Rosa Islands (85–100 %) but similar to those described in Iran, Ontario, Hungary, and Serbia, ranging from 1 to 10 % [2,34,57,63,67].

One of the factors that influences the spread of an infection in wild animals is the prevalence and incidence in domestic animals. Recent studies have established a seroprevalence of 5.97 % for *C. burnetii*, 8.2 % for HEV, 0.8 % for PRV, 16.03 % for *E. canis*, 7.8 % for *A. phagocytophilum*, and 0.2 % for *B. burgdorferi* and *D. immitis* in domestic dogs (*Canis lupus familiaris*) [14,16,17,58].

The presence of numerous endoparasite eggs was an expected result, as also highlighted in other studies. Evidence described in the literature has reported frequent *T. canis* and *Strongyloides* spp. infections in foxes and *Capillaria* spp. and *Crenosoma* spp. in hedgehogs [2,22,33,39,40,52,59]. *Crenosoma vulpis* has a wide distribution in both foxes and dogs in Italy [36,45,54]. The presence of parasite eggs (*Cystoisospora* spp. and *Strongyloides* spp.) in badger faeces has already been documented in other studies [38]. It is also expected that these parasites are similar to those seen in foxes, as these animals, in addition to sharing habitat, may sometimes share the den peacefully and therefore have extremely close contact [51]. The presence of numerous specimens of canids infected by *A. vasorum* was also an expected result since previous studies carried out in other countries highlighted prevalences ranging from 20 to 80 % [24,25]. Moreover, *A. vasorum* has already been detected in red foxes in Campania region in Italy, indicating that the pathogen is rooted in this area and poses a potential risk of transmission to dogs [64]. Currently, data regarding *D. immitis* infection in the red foxes in Italy are lacking, and the prevalence appears to be lower in northern Italy, as demonstrated in the study by Ferrara et al. [12,18]. Furthermore, in the present study, the authors demonstrated cross-reactions with the antigen of *D. immitis* in red foxes infected by *A. vasorum*. On the other hand, cross-reactions of sera from dogs infected with *A. vasorum* have already been demonstrated in commercially available test kits for *D. immitis*. However, these results require an extensive screening of the red fox population in Italy for the prevalence of *D. immitis* infection and possible cross-reactivity with *A. vasorum*.

Finally, the presence of the cardio-pulmonary nematodes, such as *A. vasorum*, *C. vulpis* and *E. aerophilus* in red foxes in Italy poses a significant threat for dogs that live in close proximity to foxes (such as hunting dogs) but also for dogs living in urban and suburban areas due to the ever-closer relationship between wild and domestic animals. Moreover, veterinary clinicians should necessarily consider these cardio-pulmonary nematodes in the differential diagnosis of respiratory syndrome in dogs. This parasite was initially (some decades ago) restricted to northern Italy, but its presence in wild and domestic animals across the peninsula implies extensive expansion and an urgent need for preventative measures (antiparasitics) [53].

Although the present study was conducted on a small scale, it provided clear indications of the spread of some infections in wildlife. The World Health Organization defines surveillance as the systematic collection, analysis, and interpretation of data, as well as the distribution of information to guide action. In order to pursue this concept as well as that of “One Health”, continuous surveillance must be applied to wildlife at different levels. In this context, wild animal rescue and rehabilitation centers serve as an ideal hub for monitoring a wide range of viral, bacterial, and parasitic diseases. Unlike other sampling procedures on wild animals (such as those used during hunting seasons, culling plans, capture plans, and so on), it is performed on live and less agitated animals, with biological material samples already collected for clinical study. It is also true that the findings of this study, if generalized to other rescue facilities, would indicate competent handling and treatment of wild animals, which are potential reservoirs of dangerous zoonoses.

## CRediT authorship contribution statement

**A. Minichino:** Writing – review & editing, Visualization, Software, Methodology, Investigation, Data curation, Conceptualization. **L. Ciuca:** Writing – review & editing, Visualization, Resources, Methodology, Investigation, Formal analysis, Data curation. **L. Dipineto:** Visualization, Supervision, Project administration, Conceptualization. **L. Rinaldi:** Visualization, Supervision, Project administration, Conceptualization. **S. Montagnaro:** Visualization, Supervision, Resources. **L. Borrelli:** Visualization, Supervision, Project administration. **A. Fioretti:** Visualization, Supervision, Conceptualization. **L.M. De Luca Bossa:** Resources, Methodology. **G. Garella:** Visualization, Resources, Methodology. **G. Ferrara:** Writing – review & editing, Writing – original draft, Visualization, Validation, Supervision, Resources, Methodology, Investigation, Data curation, Conceptualization.

## Declaration of competing interest

The authors declare that they have no known competing financial interests or personal relationships that could have appeared to influence the work reported in this paper.

## Data Availability

No data was used for the research described in the article.

## Appendix A Supplementary data

Supplementary material: Raw data regarding sampled animals. Image 1
